# The absence of NIPA2 enhances neural excitability through BK (big potassium) channels

**DOI:** 10.1111/cns.13119

**Published:** 2019-03-20

**Authors:** Na‐Na Liu, Han Xie, Wen‐Shu Xiang‐wei, Kai Gao, Tian‐Shuang Wang, Yu‐Wu Jiang

**Affiliations:** ^1^ Department of Pediatrics Peking University First Hospital Beijing China; ^2^ Beijing Key Laboratory of Molecular Diagnosis and Study on Pediatric Genetic Diseases Beijing China

**Keywords:** BK channels, epilepsy, neural excitability, *NIPA2*, zonisamide

## Abstract

**Aim:**

To reveal the pathogenesis and find the precision treatment for the childhood absence epilepsy (CAE) patients with *NIPA2* mutations.

**Methods:**

We performed whole‐cell patch‐clamp recordings to measure the electrophysiological properties of layer V neocortical somatosensory pyramidal neurons in wild‐type (WT) and *NIPA2*‐knockout mice.

**Results:**

We identified that layer V neocortical somatosensory pyramidal neurons isolated from the *NIPA2*‐knockout mice displayed higher frequency of spontaneous and evoked action potential, broader half‐width of evoked action potential, and smaller currents of BK channels than those from the WT mice. NS11021, a specific BK channel opener, reduced neuronal excitability in the *NIPA2*‐knockout mice. Paxilline, a selective BK channel blocker, treated WT neurons and could simulate the situation of *NIPA2*‐knockout group, thereby suggesting that the absence of NIPA2 enhanced the excitability of neocortical somatosensory pyramidal neurons by decreasing the currents of BK channels. Zonisamide, an anti‐epilepsy drug, reduced action potential firing in *NIPA2*‐knockout mice through increasing BK channel currents.

**Conclusion:**

The results indicate that the absence of NIPA2 enhances neural excitability through BK channels. Zonisamide is probably a potential treatment for *NIPA2* mutation‐induced epilepsy, which may provide a basis for the development of new treatment strategies for epilepsy.

## 1 INTRODUCTION

Childhood absence epilepsy (CAE) is a common type of genetic generalized epilepsies.^1^ Typical clinical manifestations include gazing, transient loss of consciousness, and sudden stop of motion, and they are resolved generally within 30 seconds.^2^, ^3^, ^4^ Electroencephalography shows generalized 2.5‐3.5 Hz spike‐and‐wave discharges. Susceptible CAE genes, such as *CACNA1H*, a Ca_v_3.2 T‐type calcium channel gene, are primarily associated with ion channels.^5^ CAE is also related to nonion channel genes, such as *NIPA2*, a highly selective magnesium transporter gene.^6^

*NIPA2* encodes nonimprinted Prader‐Willi/Angelman syndrome region protein 2.^7^ It belongs to *NIPA* family that includes four family members, namely, *NIPA1*,* NIPA2*,* NIPA3*, and *NIPA4*. *NIPA* family members are nonselective magnesium transporters except *NIPA2*,^6^ which is highly selective to the extracellular‐to‐intracellular transfer of magnesium.^8^, ^9^ *NIPA2* mutations associated with CAE have also been reported, at least in the Han Chinese population.^10^, ^11^ A previous study illustrated that loss‐of‐function *NIPA2* mutations may cause the accumulation of *NIPA2* proteins in the endoplasmic reticulum, block magnesium transport, and affect neuronal excitability.^12^

Various receptors and ion channels regulate neuronal excitability. Among them, big‐conductance potassium (BK) channels^13^, ^14^, ^15^, ^16^, ^17^ are controlled by magnesium,^15^ calcium,^18^ and membrane voltage.^19^ BK channels engage in action potential repolarization and play a key role in regulating action potential firing.^20^ Moreover, changes in BK channel functions can alter neuronal excitability. Genes encoding BK channels have two mutation types, namely, gain of function^20^, ^21^, ^22^ and loss of function.^23^, ^24^, ^25^ Both types may increase neuronal excitability.

The electrophysiological mechanism of *NIPA2* affecting neuronal excitability remains unknown. Here, we hypothesized that the dysfunction of *NIPA2* may reduce BK channel currents, and the decreased currents of BK channels enhance neuronal excitability.

## 2 MATERIALS AND METHODS

### 2.1 Preparation of the transverse brain slices

All experimental processes involving the use of animals were approved by the Experimental Animal Sciences of Peking University Health Science Center Institutional Animal Care and Use Committee. Animal pain was minimized, and the number of animals was reduced through maximal efforts. C57BL/6J *NIPA2*‐knockout mice were generated by Beijing Biocytogen Co., Ltd. The *NIPA2*‐knockout mice used were all homozygous animals. And *NIPA2*‐knockout mice inbred of six or seven generations into the C57BL/6J background were used.

Brain slices were obtained from male and female juvenile mice (age ranged from postnatal day P14 to P21). The mice were subjected to general anesthesia with chloral hydrate (0.36 g/kg, intraperitoneally) and perfused transcardially with ice‐cold oxygenated (95% O_2_ and 5% CO_2_) sucrose‐substituted artificial cerebrospinal fluid (s‐ACSF) comprising the following for 2 minutes (min): 206 mmol/L sucrose, 11 mmol/L glucose, 2.5 mmol/L KCl, 0.5 mmol/L CaCl_2_, 10 mmol/L MgCl_2_, 1.25 mmol/L NaH_2_PO_4_, and 26 mmol/L NaHCO_3_. The brain was immediately segregated and immersed in the ice‐cold oxygenated s‐ACSF. Coronal transverse slices with a thickness of 350 μm were obtained with a vibrating microslicer (VT1000S; Leica, Nussloch, Germany). The slices were incubated at 32°C for at least 30 minutes in regular oxygenated ACSF comprising the following: 126 mmol/L NaCl, 3 mmol/L KCl, 2 mmol/L CaCl_2_, 1.3 mmol/L MgSO_4_, 1.25 mmol/L NaH_2_PO_4_, 26 mmol/L NaHCO_3_, and 20 mmol/L D‐glucose (pH = 7.3‐7.4). They were stored at 22°C‐24°C.

### 2.2 Electrophysiological recordings

**Figure 1:**
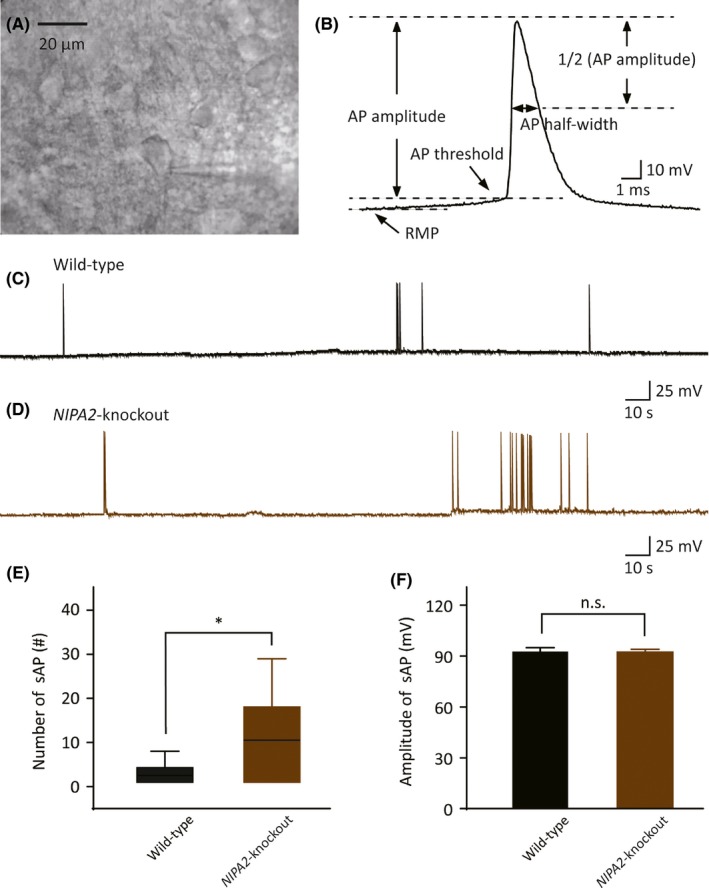
Functional characterization of spontaneous APs in the neocortical somatosensory pyramidal neurons. A, A 40× view of representative photograph of the layer V neocortical somatosensory pyramidal neurons. B, Schematic diagram of action potential. C, Representative trace of spontaneous APs in the neurons isolated from the WT mice; scale bars represent 10 s (*x*‐axis) and 25 mV (*y*‐axis). D, Spontaneous APs in the neurons isolated from the *NIPA2*‐knockout mice. Scale bars are identical to that in C. E, Number of spontaneous APs in the neurons isolated from the WT mice and the *NIPA2*‐knockout mice within 5 min. Statistical significance was obtained by Wilcoxon signed rank test. *P < 0.05. F, Amplitude of spontaneous APs in the neurons isolated from the WT mice and *NIPA2*‐knockout mice (by *t* test). APs, action potentials; n.s., no significance; sAP, spontaneous action potential; WT, wild‐type

After the brain slices were prepared, the electrophysiological properties of the whole‐cell patch‐clamp recording procedures were determined. An incubated slice was transferred to the recording chamber, placed under an upright microscope (BX51WI; Olympus), and continuously perfused with oxygenated ACSF (2‐4 mL/min). The layer V neocortical somatosensory pyramidal neurons in the brain slices could be identified with an infrared and differential interference contrast (IR‐DIC) camera (IR‐1000; Dage, Michigan City, IN). A representative photograph of these neurons is presented in the Figure 1A. The borosilicate glass (1.50 mm OD, 1.10 mm ID; Sutter Instrument, Novato, CA) was pulled into recording pipettes on a micropipette puller (PC‐10; Narishige, Japan) and filled with a solution containing the following: 134 mmol/L K‐gluconate, 3.5 mmol/L KCl, 0.1 mmol/L CaCl_2_, 1.1 mmol/L EGTA, 10 mmol/L HEPES, 10 mmol/L phosphocreatine, 4 mmol/L Mg‐ATP, and 0.3 mmol/L Li‐GTP (pH = 7.3, adjusted with KOH, 300 mOsm). Pipette resistances were set in the range of 3‐5 MΩ. The liquid junction potential was retained. After a targeted neuron was defined and sealed tightly, whole‐cell patch‐clamp recordings were performed at room temperature. Signals were amplified using an EPC‐10 amplifier with Patchmaster (HEKA Electronik, Lambrecht, Germany). Recording was rejected when series resistance was more than 50 MΩ, when series resistance was changed by more than 20%, or when the resting membrane potential (RMP) was more positive than −55 mV. Only one neocortical somatosensory pyramidal neuron was recorded per slice, and 4‐5 slices were examined per mouse.

### 2.3 Experimental protocols

In the current‐clamp mode, the spontaneous action potential (AP) of layer V neocortical somatosensory pyramidal neurons was recorded at RMP, which was measured in the current‐clamp mode with a 0 pA holding current.

In the current‐clamp mode, at the resting membrane potential, a series of positive current injections (0 pA to +140 pA with a step of +20 pA, 1 second duration) was applied to evoke a set of AP. The evoked AP and the firing pattern were recorded at each evoking potential (0 pA to +140 pA). The evoked AP frequency was determined as the spike number in 1 second. Figure 1B shows details about how to measure the amplitude and the half‐width of action potential. The AP threshold was obtained by a special voltage stimulus from −50 to −30 mV with a step of 1 mV and a duration of 100 ms, and the voltage evoking the initial current was the AP threshold.^26^

The currents of the BK channels were evoked as described previously.^27^ In the voltage‐clamp mode, under the holding potential of −80 mV, a series of depolarizing step voltage pulses (+40 mV to +180 mV with a step of +20 mV, 75 ms duration) was applied to evoke BK channel currents. At a potential of +40 mV to +180 mV, the curve of BK channel currents was the difference between that of the total currents before and after paxilline (blocking BK channels) and tetrodotoxin (TTX; blocking sodium channels) were used. The current density was calculated by dividing the corresponding amplitude of BK channel currents by the cell capacitance at each evoking potential (+40 mV to +180 mV). The current density‐voltage curve was drawn.

### 2.4 Chemical application

All chemicals were bath applied through a peristaltic pump system at a steady perfusion and suction rate. The time required for the solution to flow to the recording slice through stopcocks was approximately 1 minute. All drugs, including paxilline (a specific BK channel blocker^28^, ^29^), were purchased from Sigma‐Aldrich (St. Louis, MO, USA), except NS11021 (a specific BK channel opener^27^, ^30^, ^31^) and TTX (a sodium channel blocker), which were bought from Tocris Bioscience (Bristol, UK). All chemicals were dissolved in dimethyl sulfoxide (except TTX that was dissolved in distilled water) at 1000 times final concentration, stored at −20°C, and diluted to the final concentration in an oxygenated ASCF solution immediately before use.

### 2.5 Statistical analysis

All numerical data were expressed as mean ± SEM, except abnormal distribution data, which were presented as the median ± quartile. Unless otherwise mentioned, n refers to the recorded number of layer V neocortical somatosensory pyramidal neurons. The frequency and amplitude of spontaneous AP were analyzed offline with MiniAnalysis 6.0.3 (Synaptosoft, Inc, Decatur, GA). The frequency and amplitude of the evoked AP, and BK currents were examined offline with Clampfit 10.1 (Axon Instruments) and Igor (WaveMetrics, Inc, Portland, OR). All statistical analyses were performed on SPSS version 24.0 (SPSS, Chicago, IL). Wilcoxon signed rank test was used for the abnormal distribution data. When conforming to a normal distribution, the statistical significance of the data from two independent groups or different chemical conditions was assessed with paired or unpaired Student's *t* tests. Statistical significance was indicated as *P < 0.05, **P < 0.01, and ***P < 0.001.

## 3 RESULTS

### 3.1 Neocortical somatosensory pyramidal neurons of *NIPA2*‐knockout mice showed excessive excitability

*NIPA2* mutations have been observed in children with CAE, resulting in decreased intracellular magnesium concentrations.^10^, ^12^ However, whether changes in NIPA2 function enhance neuronal excitability remains unknown.

Magnesium is a vital regulatory factor of many ion channels, such as BK channels, associated with neuronal excitability. To investigate the effect of *NIPA2* mutations on neuronal excitability, we performed whole‐cell current‐clamp recordings between the two independent groups, namely, the wild‐type (WT) mouse group and the *NIPA2‐*knockout mouse group. In the current‐clamp mode, we measured spontaneous and evoked APs of the layer V neocortical somatosensory pyramidal neurons isolated from WT or *NIPA2*‐knockout mice. The neurons isolated from the WT mice generated minor or minimal spontaneous APs within 5 minutes. Correspondingly, the frequency of the spontaneous APs of the neurons isolated from the *NIPA2*‐knockout mice was significantly higher than that from the WT mice (Figure 1B‐E, WT: 1.5 ± 1.0, n = 10 vs *NIPA2* knockout: 4.0 ± 10, n = 10, *P* = 0.038). The amplitude of spontaneous APs had no difference between the two groups (Figure 1F, WT: 93 ± 2.2 mV vs *NIPA2* knockout: 92 ± 1.7 mV, *P = *0.90).

**Figure 2:**
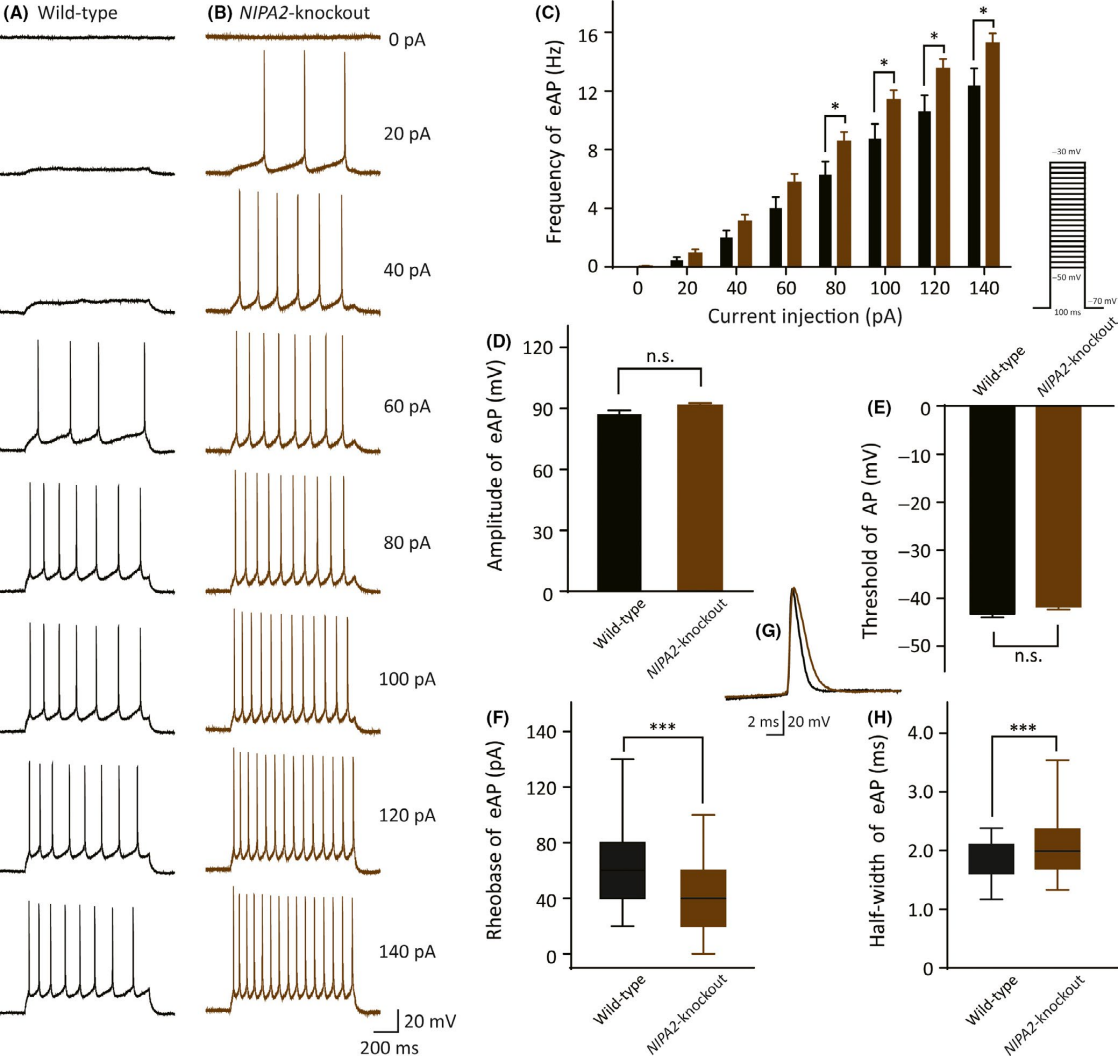
Functional characterization of evoked APs in the neocortical somatosensory pyramidal neurons. A, Exemplar traces of evoked APs in the WT neurons in response to test currents ranging from 0 pA to 140 pA with a step of 20 pA and duration of 1 s. Scale bars are identical to that in B. B, Recorded evoked APs under the same stepped current injections with A in *NIPA2*‐knockout neurons. Scale bars are 200 ms (*x*‐axis) and 20 mV (*y*‐axis). C, Frequency of evoked APs in response to the current injections in the neurons isolated from the WT mice (black, n = 37) and the *NIPA2*‐knockout mice (brown, n = 60). The neurons from *NIPA2*‐knockout mice had higher frequency of evoked APs at current injections of 80‐140 pA (by *t* tests separately). *P < 0.05. D, Amplitude of evoked first AP responded to 80 pA current stimulus in the WT and the *NIPA2*‐knockout neocortical pyramidal neurons. E, Threshold of action potential in the WT and the *NIPA2*‐knockout neurons. The protocol to determine the AP threshold is presented above. F, Rheobase of evoked initial APs in the WT and the *NIPA2*‐knockout neurons. Statistical significance was obtained by Wilcoxon signed rank test. ***P < 0.001. G, Sample traces of first firing responded to 80 pA stimulus in the WT (black) and the *NIPA2*‐knockout (brown) neurons. Scale bars represent 2 ms (*x*‐axis) and 20 mV (*y*‐axis). H, Evoked APs half‐width at the beginning of the AP trace by an 80 pA current stimulus in the WT and the *NIPA2*‐knockout neurons. Statistical significance was obtained by Wilcoxon signed rank test. ***P < 0.001. APs, action potentials; eAP, evoked action potential; WT, wild‐type

We also measured the evoked AP firing activities in the layer V neocortical somatosensory pyramidal neurons and found that the frequency of the evoked APs of the neurons isolated from the *NIPA2*‐knockout mice was significantly higher than that of the neurons from the WT mice through a train of current injection (Figure 2). The neurons from the *NIPA2*‐knockout mice were more excitable at current injections of 80, 100, 120, and 140 pA and showed a sustained activity at high current injections, such as 80 pA (Figure 2A‐C, WT: 6.3 ± 0.90 Hz, n = 37 vs *NIPA2* knockout: 8.6 ± 0.65 Hz, n = 60, *P = *0.041). Differences were observed between the two groups in terms of RMP (WT: −65 ± 0.56 mV, n = 37 vs *NIPA2* knockout: −62 ± 0.46 mV, n = 60, *P = *0.000, not shown). And no differences were observed in termed of evoked AP amplitude (Figure 2D, WT: 87 ± 1.9 mV, n = 23 vs *NIPA2* knockout: 91 ± 1.2 mV, n = 55, *P = *0.057) and AP threshold (Figure 2E, WT: −43 ± 0.70 mV, n = 35 vs *NIPA2* knockout: −42 ± 0.55 mV, n = 60, *P = *0.11). The AP rheobase (the smallest injected current evoking first AP) of the neurons of the *NIPA2*‐knockout mice was lower than that of the WT mice (Figure 2F, WT: 60 ± 40 pA, n = 35 vs *NIPA2* knockout: 40 ± 40 pA, n = 60, *P* = 0.000). The neurons from the *NIPA2*‐knockout mice exhibited a broad evoked AP half‐width at the beginning of the AP trace evoked by an 80 pA current stimulus (Figure 2G‐H, first evoked AP: WT: 1.8 ± 0.49 ms, n = 23 vs *NIPA2* knockout: 2.1 ± 0.68 ms, n = 55, *P* = 0.000). These results indicated that the neocortical somatosensory pyramidal neurons of the *NIPA2*‐knockout mice obtained excessive excitability. The difference in the evoked AP half‐width between the two groups suggested that slowing the outflow of potassium during AP repolarization might be associated with increased neuronal excitability in the *NIPA2*‐knockout mice.

### 3.2 Absence of NIPA2 reduced the currents of BK channels

**Figure 3:**
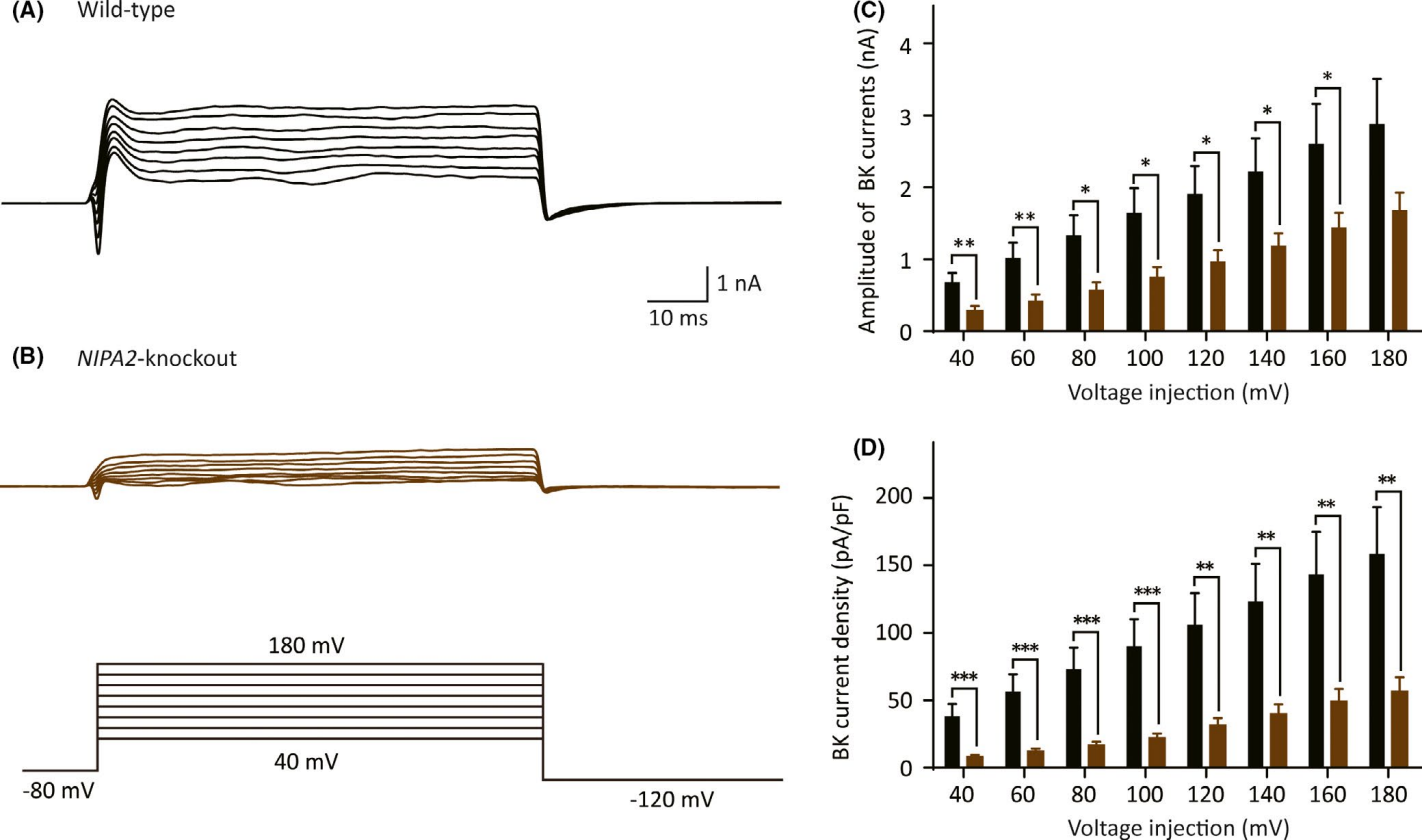
Functional characterization of BK channel currents in the neocortical somatosensory pyramidal neurons. A, Traces of BK channel currents in the WT neurons. Scale bars represent 10 ms (*x*‐axis) and 1 nA (*y*‐axis). B, Traces of BK channel currents in the *NIPA2*‐knockout neurons. Scale bars are identical to that in A. The protocol to record the BK channel currents is presented at the bottom. C, Amplitude of BK channel currents in the WT (black, n = 8) and the *NIPA2*‐knockout neurons (brown, n = 13). The neurons from the *NIPA2*‐knockout mice had smaller BK channel currents in response to voltage injections of 40‐160 mV. Statistical significance was obtained by *t* test separately. D, Plots of BK current density against the test voltages. The *NIPA2*‐knockout neurons had lower current density of 40‐180 mV than the WT neurons. *P < 0.05, **P < 0.01, ***P < 0.001. WT, wild‐type

The difference in the evoked AP half‐width between the WT group and the *NIPA2*‐knockout group suggested that potassium channels might be involved in NIPA2‐regulated neural excitability. BK channels were activated by intracellular magnesium and played a vital role in AP repolarization. NIPA2 dysfunction could decrease intracellular magnesium concentrations. Thus, we inferred that the absence of NIPA2 might enhance neuronal excitability by decreasing BK channel currents. In our study, after 0.5 μmol/L TTX were used at least 5 minutes to block sodium channels, 10 μmol/L paxilline was provided for 10 minutes in 0.5 μmol/L TTX‐containing bath to completely inhibit the BK channel currents, and the maximum inhibitory effects were measured 1‐2 minutes after the end of paxilline and TTX bath perfusion. The amplitude of the BK currents was measured as the difference between the total currents after a +180 mV voltage step and at an initial holding potential of +40 mV. We found that the BK channel currents of the neurons isolated from the *NIPA2*‐knockout mice were significantly smaller than those of the neurons from the WT mice at voltage injections of 40‐160 mV (Figure 3A‐C), although no significance was observed at +180 mV stimulus (WT: 2.9 ± 0.63 nA, n = 8 vs *NIPA2* knockout: 1.7 ± 0.24 nA, n = 13, *P = *0.062). The current density was obtained by dividing the amplitude of the BK channel currents at each voltage injection by the cell capacitance. A significant decrease was observed in the *NIPA2*‐knockout group in terms of current density compared with that in the WT group (Figure 3D, WT: 159 ± 35 pA/pF, n = 8 vs *NIPA2* knockout: 57 ± 10 pA/pF, n = 13, *P = *0.0031). These data revealed that the absence of NIPA2 decreased the BK channel currents, possibly hindering AP repolarization and contributing to the increased spontaneous and evoked AP firing frequency.

### 3.3 Increased BK channel currents reduced action potential firing in *NIPA2*‐knockout mice

**Figure 4:**
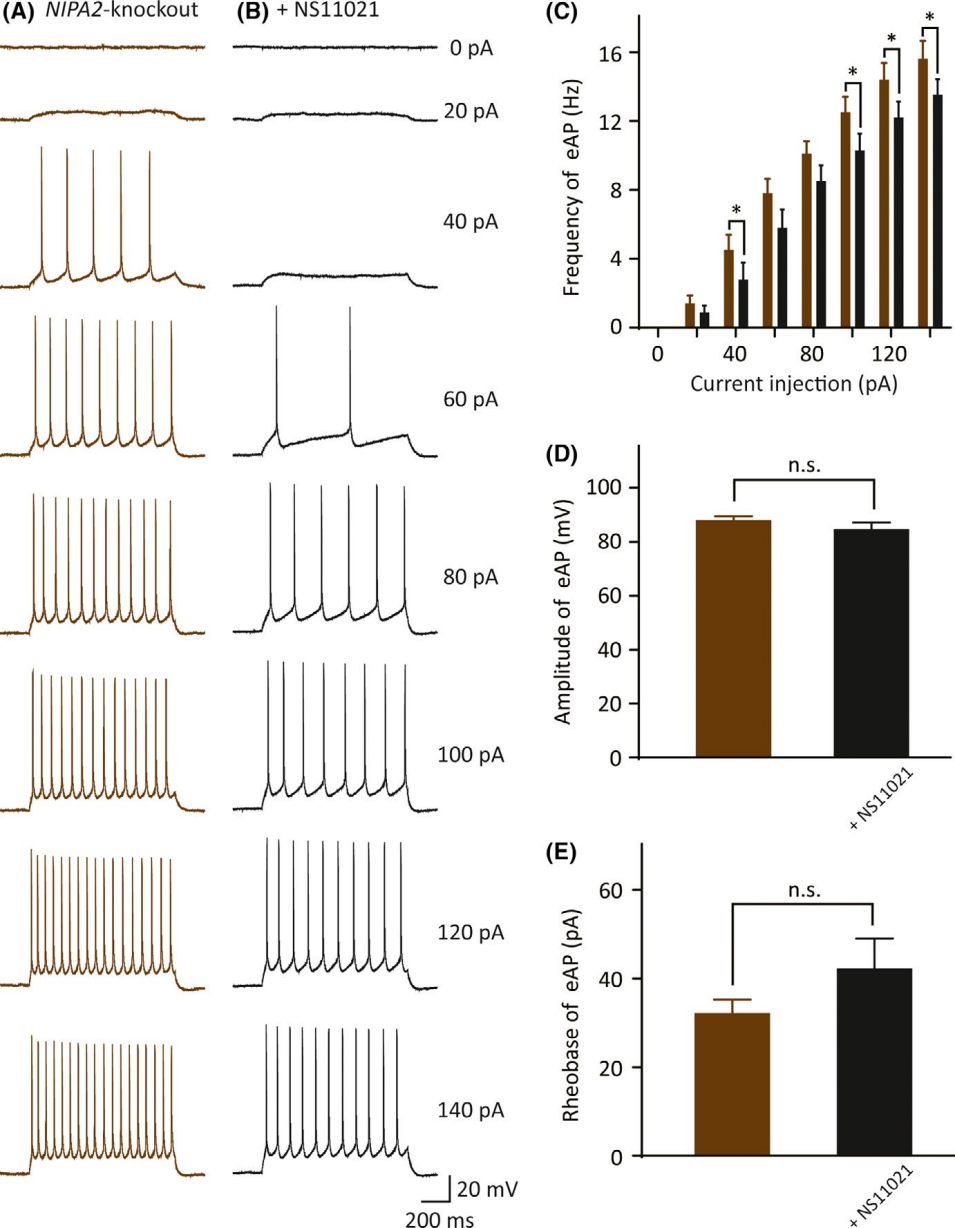
Evoked AP changes in *NIPA2*‐knockout neurons after the use of BK channel opener. A, Exemplar traces of evoked APs in *NIPA2*‐knockout neurons in response to test currents ranging from 0 pA to 140 pA in 20 pA increments for 1 s in the absence of NS11021, a selective BK channel opener. Scale bars are identical to that in B. B, Recorded evoked APs under the same test current injections with A in *NIPA2*‐knockout neurons in the presence of NS11021. Scale bars represent 200 ms (*x*‐axis) and 20 mV (*y*‐axis). C, Frequency of evoked APs in response to the current injections in the neurons without NS11021 (brown, n = 10) or with NS11021 (black, n = 10). The neurons without NS11021 exhibited higher evoked APs at current injections of 40, 100, 120, and 140 pA. Statistical significance was obtained by *t* test separately. *P < 0.05. D, Amplitude of evoked first AP responded to 80 pA current stimulus without or with NS11021. E, Rheobase of evoked initial APs in the neurons without or with NS11021. APs, action potentials

To demonstrate whether the increased BK channel currents in the *NIPA2‐*knockout mice could decrease neuronal excitability, we compared the AP firing trains derived from a range of current injection in the *NIPA2*‐knockout mice in the absence or presence of the BK channel opener NS11021. We perfused 3 μmol/L NS11021 for 10 minutes to completely activate BK currents and recorded the evoked AP firing across a train of 0‐140 pA current injections every 2 minutes from the initial NS11021 usage to 15 minutes after the end of NS11021 perfusion. For example, in the current stimulus of 140 pA, the frequency of the evoked AP firing of the neocortical somatosensory pyramidal neurons in the *NIPA2*‐knockout mice was significantly decreased after NS11021 was added (Figure 4A‐C, without NS11021: 16 ± 1.1 Hz, n = 10 vs with NS11021: 14 ± 0.91 Hz, n = 10, *P = *0.029). At the same current stimulus, in the *NIPA2*‐knockout mice with or without the application of the BK channel opener, no differences were observed in the evoked AP amplitude (Figure 4D, without NS11021: 88 ± 1.8 mV vs with NS11021: 84 ± 2.8 mV, *P = *0.052) and the evoked AP rheobase (Figure 4E, without NS11021: 30 ± 3.3 pA vs with NS11021: 42 ± 7.0 pA, *P = *0.051). These results suggested that the decrease in the BK channel currents was the main reason for the excessive excitability of neocortical somatosensory pyramidal neurons of the *NIPA2*‐knockout mice.

### 3.4 Decreased BK channel currents enhanced action potential firing in wild‐type mice

**Figure 5:**
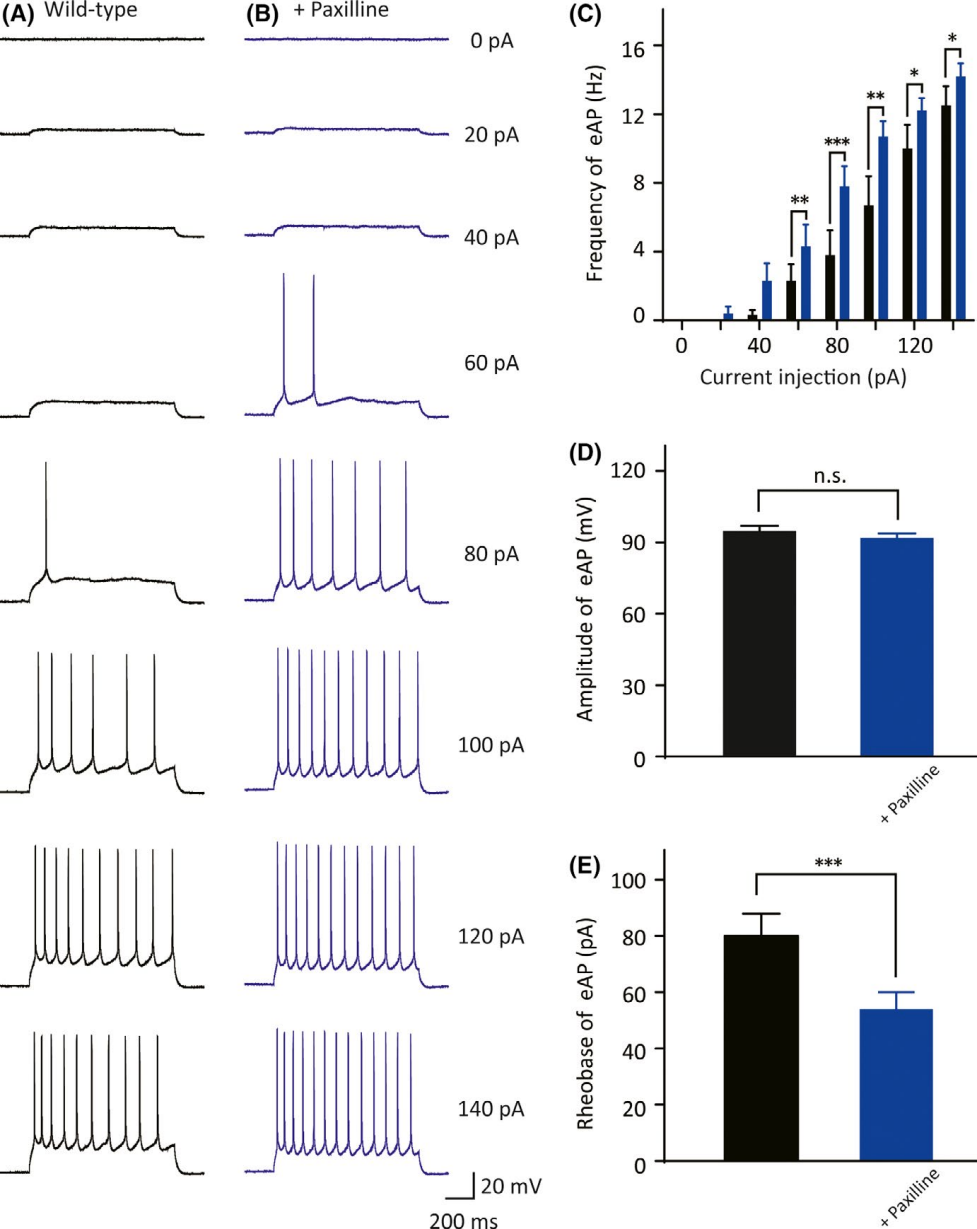
Evoked AP changes in the WT neurons after the use of BK channel blocker. A, Exemplar traces of evoked APs in the WT neurons in response to test currents ranging from 0 pA to 140 pA in 20 pA increments for 1 s in the absence of paxilline, a selective BK channel blocker. Scale bars are identical to that in B. B, Recorded evoked APs under the same test current injections with A in the WT neurons in the presence of paxilline. Scale bars represent 200 ms (*x*‐axis) and 20 mV (*y*‐axis). C, Frequency of evoked APs in response to the current injections in the neurons without paxilline (black, n = 10) or with paxilline (blue, n = 10). The neurons without paxilline exhibited lower frequency of evoked APs at current injections of 60‐140 pA. Statistical significance was obtained by *t* test separately. *P < 0.05, **P < 0.01, ***P < 0.001. D, Amplitude of evoked first AP responded to 80 pA current stimulus without paxilline or with paxilline. E, Rheobase of evoked initial APs in the neurons without paxilline or with paxilline. Statistical significance was obtained by *t* test. ***P < 0.001. APs, action potentials; WT, wild‐type

To further observe the effect of BK channels on AP, we added 10 μmol/L paxilline for 10 minutes to block the currents of the BK channels of the WT mice, thereby simulating the situation of *NIPA2*‐knockout mice. We compared the AP firing trains derived from a range of current injection in the WT mice in the absence or presence of the BK channel blocker paxilline. At the 140 pA current stimulus, the frequency of the evoked AP firing of the neocortical somatosensory pyramidal neurons of the WT mice displayed was significantly high after paxilline was added (Figure 5A‐C, without paxilline: 13 ± 1.1 Hz, n = 10 vs with paxilline: 14 ± 0.77 Hz, n = 10, *P = *0.045). The rheobase of the evoked AP declined significantly after paxilline was added (Figure 5E, without paxilline: 80 ± 7.9 pA vs with paxilline: 54 ± 6.0 pA, *P = *0.00075). However, the evoked AP amplitude was not significantly changed by paxilline (Figure 5D, without paxilline: 95 ± 2.3 mV vs with paxilline: 92 ± 1.9 mV, *P = *0.11). These results illustrated that decreased BK channel currents enhanced AP firing in WT mice.

### 3.5 Zonisamide reduced action potential firing in *NIPA2*‐knockout mice

**Figure 6:**
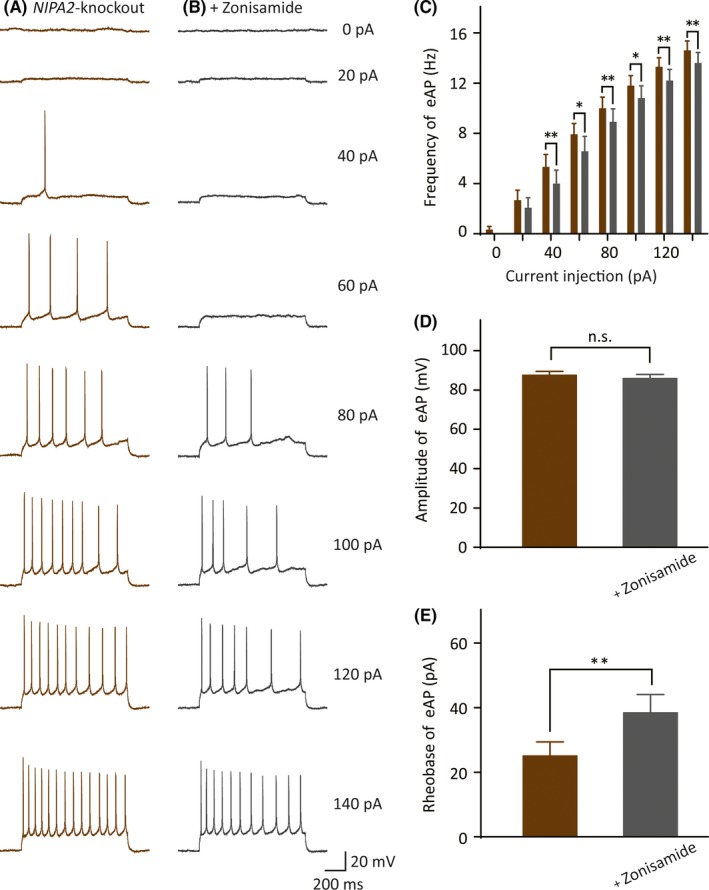
Evoked AP changes in *NIPA2*‐knockout neurons after the use of zonisamide. A, Exemplar traces of evoked APs in *NIPA2*‐knockout neurons in response to test currents ranging from 0 to 140 pA in 20 pA increments for 1 s in the absence of zonisamide. Scale bars are identical to that in B. B, Recorded evoked APs under the same test current injections with A in *NIPA2*‐knockout neurons in the presence of zonisamide. Scale bars represent 200 ms (*x*‐axis) and 20 mV (*y*‐axis). C, Frequency of evoked APs in response to the current injections in the neurons without zonisamide (brown, n = 12) or with zonisamide (silvery gray, n = 12). The neurons without zonisamide exhibited higher evoked APs at current injections from 40 to 140 pA. Statistical significance was obtained by *t* test separately. *P < 0.05, **P < 0.01. D, Amplitude of evoked first AP responded to 80 pA current stimulus without zonisamide or with zonisamide. E, Rheobase of evoked initial APs in the neurons without zonisamide or with zonisamide. **P < 0.01. APs, action potentials

Previous study showed that an antiepileptic drug of zonisamide had an anticonvulsant effect by activating BK channels.^32^ In our study, brain slices of *NIPA2*‐knockout mice were treated by zonisamide to observe neural excitability after the treatment. Specifically, we perfused 10 μmol/L zonisamide for 10 minutes to completely activate BK currents and recorded the evoked AP firing across a train of 0‐140 pA current injections every 2 minutes from the initial zonisamide usage to 10 minutes after the end of zonisamide perfusion. For example, in the current stimulus of 140 pA, the frequency of the evoked AP firing of the neocortical somatosensory pyramidal neurons in the *NIPA2*‐knockout mice was significantly decreased after zonisamide was added (Figure 6A‐C, without zonisamide: 15 ± 0.75 Hz, n = 12 vs with zonisamide: 14 ± 0.84 Hz, n = 12, *P = *0.039). At the same current stimulus, in the *NIPA2*‐knockout mice with or without the application of zonisamide, no differences were observed in the evoked AP amplitude (Figure 6D, without zonisamide: 87 ± 2.0 mV vs with zonisamide: 86 ± 2.1 mV, *P = *0.091). The rheobase of the evoked AP increased significantly after zonisamide was added (Figure 6E, without zonisamide: 25 ± 4.4 pA vs with zonisamide: 38 ± 5.8 pA, *P = *0.0046). These results suggested that zonisamide, an anticonvulsant drug increasing the BK channel currents, could inhibit the excessive excitability of neocortical somatosensory pyramidal neurons in *NIPA2*‐knockout mice.

## 4 DISCUSSION

### 4.1 Absence of NIPA2 induces neural hyperexcitability

We demonstrated that the layer V neocortical somatosensory pyramidal neurons isolated from the *NIPA2*‐knockout mice showed significantly excessive neuronal excitability. The spontaneous and evoked AP frequency of the neocortical somatosensory pyramidal neurons of the *NIPA2*‐knockout mice was higher than the WT mice. In WAG/Rij rats of absence epilepsy model, the somatosensory pyramidal neurons of the cortex with low hyperpolarization‐activated cation currents exhibit high frequency in response to current injections.^33^ For CAE, the cortico‐thalamo‐cortical network oscillation plays an important role in the occurrence and development of absence epilepsy.^34^ Abnormal discharges of CAE originate from the thalamocortical network and are finally projected into the neocortical somatosensory pyramidal neurons. Furthermore, neocortical somatosensory pyramidal neurons are a key part of the neurological circuit of absence epilepsy,^33^ and abnormal discharges of neocortical somatosensory pyramidal neurons are the feature of absence epilepsy.^35^ In the present study, the neocortical somatosensory pyramidal neurons isolated from the *NIPA2*‐knockout mice showed significantly excessive neuronal excitability. Thus, the absence of NIPA2 induced the hyperexcitability of neocortical somatosensory pyramidal neurons, thereby affecting the thalamocortical network to trigger absence epilepsy.

### 4.2 Absence of NIPA2 enhances neuronal excitability by decreasing BK channel currents

Neuronal excitability is regulated by various receptors and ion channels. Among them, BK channels and N‐methyl‐D‐aspartic acid receptor (NMDAR) channels^36^ are the two common magnesium‐regulated ones.

In this study, we found that the absence of NIPA2 enhanced neuronal excitability by decreasing BK channel currents. The BK channel currents of the layer V neocortical somatosensory pyramidal neurons of the *NIPA2*‐knockout mice were smaller than those of the WT mice. BK channels are activated by intracellular calcium and magnesium and implicated in AP repolarization. In our previous study, *NIPA2* loss‐of‐function mutation can reduce intracellular magnesium concentrations.^12^ Low magnesium concentrations decrease BK channel currents; as such, *NIPA2*‐knockout mice have decreased BK channel currents. BK channels transfer intracellular potassium to extracellular fluid for repolarization. Decreased BK channel currents may hinder repolarization and increase the frequency of AP. Therefore, the absence of NIPA2 may enhance neuronal excitability by decreasing BK channel currents. To confirm this hypothesis, we used NS11021, a specific BK channel opener, to treat the neurons of the *NIPA2*‐knockout mice and observed that it decreased the excitability of the neocortical somatosensory pyramidal neurons of the *NIPA2*‐knockout mice. We also applied paxilline to treat the neurons of the WT mice and founded that the blocker could enhance neuronal excitability in the WT mice. These findings further suggested that the absence of NIPA2 improved neuronal excitability through the BK channels.

An increase in BK channel currents usually leads to absence epilepsy,^37^, ^38^ and a decrease in BK channel currents may be associated with temporal lobe epilepsy.^23^ However, we identified *NIPA2* loss‐of‐function mutations from patients with CAE, and NIPA2 dysfunction can decrease BK channel currents to obtain neuronal hyperexcitability. Our results suggested that the decrease in BK channel currents may also participate in the onset of absence epilepsy.

In addition to BK channels, changes of magnesium concentration may affect NMDAR channels. Previous studies have reported that intracellular magnesium blocks the activation of NMDAR channels to modulate synaptic strength.^39^, ^40^ In addition, our previous studies have demonstrated that dysfunction of NIPA2 brings out decreased intracellular magnesium concentration.^12^ Thus, we hypothesize that currents through NMDAR channels may increase in the *NIPA2*‐knockout mice; however, more studies are needed to verify this hypothesis.

### 4.3 Zonisamide is probably a potential treatment for epilepsy patients with *NIPA2* loss‐of‐function mutation

Precision medicine has been extensively used to treat patients with epilepsy. For example, quinidine can be applied to treat *KCNT1*‐positive epilepsy,^41^, ^42^ and retigabine is utilized for *KCNQ2*‐positive epilepsy.^43^, ^44^ We suggested that zonisamide might be a precision treatment for epilepsy patients with *NIPA2* loss‐of‐function mutation. We identified three *NIPA2* mutations (c.532A > T, c.731A > G, c.1002_1003insGAT) from children with CAE.^10^, ^11^ However, we did not find *NIPA2* variations in children with epilepsy and intellectual/developmental disabilities through targeted next‐generation sequencing or Sanger sequencing.^45^ A subsequent functional study has verified that these three mutations are loss of function.^12^ In the present study, the dysfunction of NIPA2 enhanced the excitability of neocortical somatosensory pyramidal neurons by decreasing BK channel currents. *NIPA2* loss‐of‐function mutations might serve as pathogenic mutations in children with CAE. If a drug could rescue *NIPA2*‐induced pathogenic process, then this drug would save patients with epilepsy. Zonisamide, as a commercially available antiepileptic drug, elicits an anticonvulsant effect by activating BK channels, and it reduces neuronal hyperexcitability by increasing BK channel currents.^32^ Therefore, this drug might be used to treat the epilepsy patients with *NIPA2* loss‐of‐function mutation. Previous preclinical studies on zonisamide emphasized that its biological metabolism is prototype metabolism without drug attenuation. In this study, brain slices of *NIPA2*‐knockout mice were treated by zonisamide, and we have found that zonisamide reduce action potential firing in *NIPA2*‐knockout mice. It suggests that zonisamide may be an effective treatment for *NIPA2*‐knockout mice. We believe that zonisamide is probably a potential treatment for *NIPA2* mutation‐induced epilepsy.

In summary, the dysfunction of NIPA2 enhances neuronal excitability by decreasing BK channel currents. *NIPA2* loss‐of‐function mutations are pathogenic in patients with CAE. Zonisamide may be used to treat the epilepsy patients with *NIPA2* loss‐of‐function mutation.

## 5 CONCLUSION

In this study, we found that the absence of NIPA2 enhanced the excitability of neocortical somatosensory pyramidal neurons by decreasing the currents of BK channels. Zonisamide, reducing action potential firing in *NIPA2*‐knockout mice, is probably a potential treatment for *NIPA2* mutation‐induced epilepsy, which may provide a basis for the development of new treatment strategies for epilepsy.

## CONFLICT OF INTEREST

The authors declare no conflict of interest.
